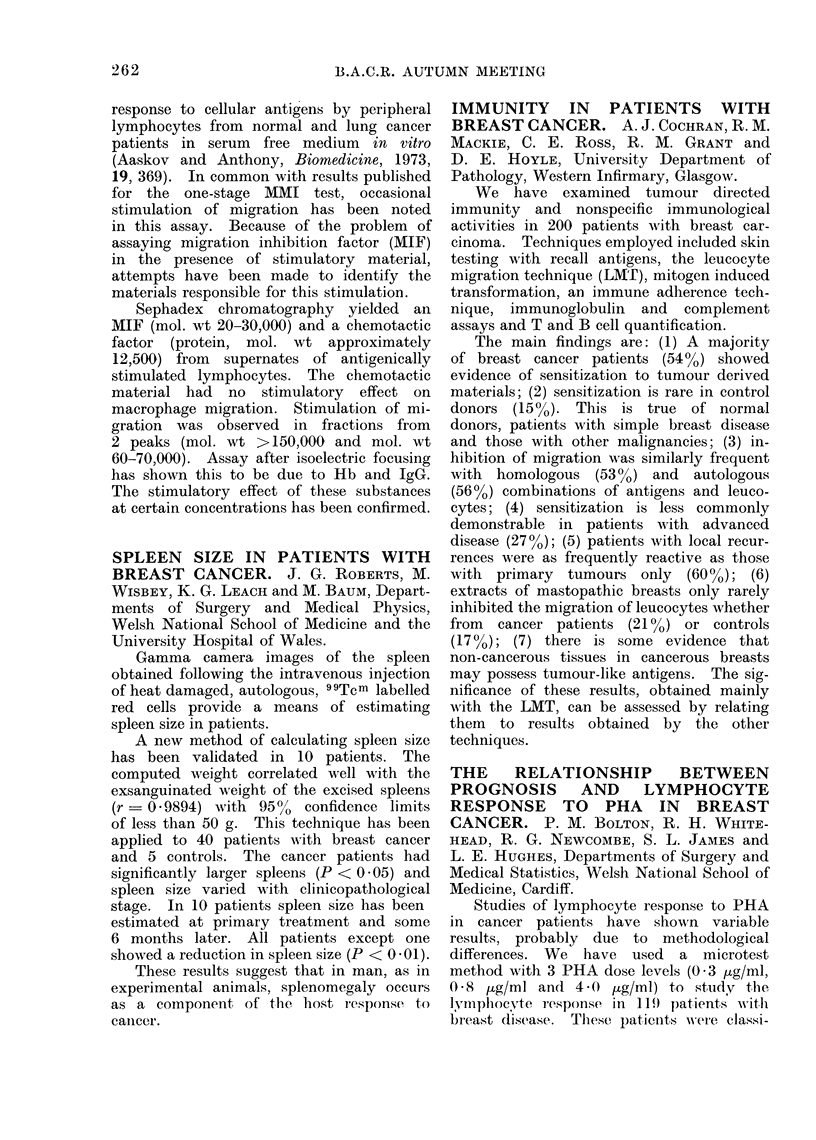# Proceedings: Spleen size in patients with breast cancer.

**DOI:** 10.1038/bjc.1975.44

**Published:** 1975-02

**Authors:** J. G. Roberts, M. Wisbey, K. G. Leach, M. Baum


**IMMUNITY IN PATIENTS WITH BREAST CANCER.** A. J. Cochran, R. M. Mackie, C. E. Ross, R. M. Grant and D. E. Hoyle, University Department of Pathology, Western Infirmary, Glasgow.

We have examined tumour directed immunity and nonspecific immunological activities in 200 patients with breast carcinoma. Techniques employed included skin testing with recall antigens, the leucocyte migration technique (LMT), mitogen induced transformation, an immune adherence technique, immunoglobulin and complement assays and T and B cell quantification.

The main findings are: (1) A majority of breast cancer patients (54%) showed evidence of sensitization to tumour derived materials; (2) sensitization is rare in control donors (15%). This is true of normal donors, patients with simple breast disease and those with other malignancies; (3) inhibition of migration was similarly frequent with homologous (53%) and autologous (56%) combinations of antigens and leucocytes; (4) sensitization is less commonly demonstrable in patients with advanced disease (27%); (5) patients with local recurrences were as frequently reactive as those with primary tumours only (60%); (6) extracts of mastopathic breasts only rarely inhibited the migration of leucocytes whether from cancer patients (21%) or controls (17%); (7) there is some evidence that non-cancerous tissues in cancerous breasts may possess tumour-like antigens. The significance of these results, obtained mainly with the LMT, can be assessed by relating them to results obtained by the other techniques.